# Social media as a public health tool during the UK mpox outbreak: a qualitative study of stakeholders’ experiences

**DOI:** 10.1136/bmjph-2023-000407

**Published:** 2023-10-25

**Authors:** Jaime Garcia-Iglesias, Tom May, Martyn Pickersgill, Jeremy Williams, Maurice Nagington, Sophie Buijsen, Ciara McHugh, Jeremy Horwood

**Affiliations:** 1 Centre for Biomedicine, Self and Society, Usher Institute, The University of Edinburgh, Edinburgh, UK; 2 NIHR Health Protection Research Unit in Behavioural Science and Evaluation, University of Bristol, Bristol, UK; 3 International Public Policy Observatory, UCL, London, UK; 4 School of Health Sciences, University of Manchester, Manchester, UK; 5 Science, Technology and Innovation Studies, School of Social and Political Science, The University of Edinburgh, Edinburgh, UK; 6 Mitchell Institute for Global Peace, Security and Justice, Queen's University Belfast, Belfast, UK

**Keywords:** Public Health, Disease Outbreaks, Communicable Disease Control

## Abstract

**Objectives:**

This rapid response research explored the experiences of key stakeholder groups during the 2022–2023 mpox outbreak in the UK, and in particular, the use of social media as a tool of health promotion. The project sought to identify key lessons learnt for future epidemic and pandemic preparedness.

**Methods:**

The qualitative study employed online focus groups and interviews with key stakeholder communities, including activists, clinicians, policy actors and individuals with lived experience of mpox. N=29 stakeholders participated. Data were subject to framework analysis, with findings discussed and conclusions reached through a face-to-face analysis workshop.

**Results:**

Participants emphasised the significant role of social media, particularly Twitter (now called X), in the response to the mpox outbreak. Several benefits were highlighted, including disseminating relevant information, tackling stigma and generating/advancing advocacy and collaboration. However, participants also pointed out challenges associated with social media; in particular, its reliance on pre-existing networks and associated dynamics of exclusion, and the presence of misinformation.

**Conclusion:**

Social media played an important role in informal and purposive health promotion during the 2022–2023 mpox outbreak, while also presenting significant challenges regarding misinformation and exclusion. We recommend that preparedness for infectious disease outbreaks must consider the role of social media as key tools for not only the dissemination of health promotion messages, but also for real-time collaboration on message development. Special attention should be paid to ensuring collaboration and dissemination strategies are explicitly orientated towards promoting the inclusion of underserved groups.

**WHAT IS ALREADY KNOWN ON THIS TOPIC**

- Social media has been recognised as an important tool for health promotion with key benefits (eg, supporting communities, fostering collaboration or allowing advocacy). At the same time, social media can also disseminate misleading or inaccurate information, including from trusted voices.

**WHAT THIS STUDY ADDS**

- This study evidences that social media allowed for swift dissemination of relevant health promotion and vaccination information, for people to tackle stigma around contracting mpox, and for different stakeholders to collaborate in developing and sharing messaging.
- Our participants also explained that social media posed important problems: it relied on pre-existing networks, generated exclusion and commonly contained inaccurate or misleading information.

**HOW THIS STUDY MIGHT AFFECT RESEARCH, PRACTICE OR POLICY**

- Social media offers an opportunity for asynchronous yet rapid collaboration and cocreation of health promotion strategies and messages with community organisations.
- Despite the advantages of platforms such as Twitter, health communication strategies should leverage a range of social media, alongside more traditional modes of messaging, to help avoid forms of digital exclusions.

## Introduction

 Mpox, formerly known as monkeypox, is a disease caused by an orthopoxvirus—the mpox virus. Endemic to Central and West Africa, since May 2022 non-endemic countries have experienced mpox outbreaks. On 23 July 2022, the WHO declared the disease a Public Health Emergency of International Concern (PHEIC).^1^ As of September 2023, over 90 000 cases have been confirmed worldwide across 111 countries, with the USA, Brazil, Spain and France, Colombia, Mexico, Peru and UK experiencing the majority of cases.

The 2022–2023 mpox outbreak affected primarily men who have sex with men (MSM; 84.1%), and sexual encounters were the most common type of reported transmission (82%).^2^ This has led scholars to suggest that mpox, in the 2022 outbreak, is a de facto sexually transmitted infection.^3 4^ In the UK, public health authorities rapidly identified gay and bisexual MSM as communities with high exposure risk to mpox. Vaccine access for them was subsequently prioritised. Despite this, vaccination was not always readily accessible.^5^

On December 2022, the UK Government released a national ‘public health strategy for controlling mpox’. This aimed to reduce harm, suppress UK transmission, minimise imported cases and reduce the global burden. Among the categories of interventions considered key for meeting these goals, the UK Health Security Agency (UKHSA) recommended ‘community engagement and risk communication’. The strategy called for communications to target at-risk populations (especially underserved ones), focusing on vaccination, minimising stigma and engaging with organisations to ‘refine messaging’.^6^ This is in the context of an increased use of social media to disseminate public health messages.

The effective use of social media for health promotion has been described as simultaneously a ‘unique opportunity for public health’^7^ and the ‘defining public health challenge of this century’.^8^ In the UK, there is ample experience of the importance of social media in health promotion and public health gained through long-term HIV promotion (such as PrEP--pre-exposure prophylaxis for HIV--and U=U) and, most recently, COVID-19.^9^,^11^ There are several benefits to the inclusion of social media in public health strategies: social media may foster collaboration and engagement between different stakeholders, support communities (especially around stigmatised conditions), serve as a platform for advocacy, and allow for the sharing of personal stories.^12^,^16^ However, scholars have also identified important challenges. Most notably, Stellefson *et al* argue that social media are ‘a wild west for health information’^12^ where users can freely interact with both accurate and inaccurate information, and Schillinger *et al* consider online platforms as venues for ‘infodemics’,^11^ or the availability of ‘too much information, including false or misleading information’.^17^ Overall, there is no clear agreement that supports the universal ‘effectiveness of social media to improve public health outcomes and trends’.^12^

This paper explores experiences, benefits and challenges of the use of social media in the 2022–2023 mpox outbreak. It reports on qualitative data gathered through focus groups and interviews with a range of key stakeholder communities across the UK and the USA. It asks: what are key stakeholders’ experiences of using social media as an informal or explicit health promotion tool during the mpox epidemic in the UK, and what lessons can be learnt for future epidemic/pandemic preparedness and management?

## Methods

### Design and setting

A qualitative study was conducted based in a series of three focus groups and ten semistructured interviews with key stakeholder communities, which were defined as: (1) activists and people from third sector organisations involved in the mpox response, (2) clinicians and those working in medical settings related to mpox, (3) policy actors and others involved in local or national governmental responses and (4) individuals with lived experience of mpox (LEM). While the emphasis of the project was on the UK context, participants from the USA were also invited to the clinicians’, policy actors’ and activists’ groups to help illustrate where and how responses and experiences in the UK could have been otherwise. One individual with LEM who participated in an interview was based in Germany.

### Recruitment

The research team included academics with a background in policy, sociology, public health and nursing, who used their existing networks within sexual health to launch snowball sampling recruitment by disseminating invitations to eligible participants via email. Social media (Twitter)^1^ was also used to advertise the study among people with LEM. Recruitment was performed in this way due to the rapid nature of the project, which allowed for only two weeks for recruitment. Emphasis was placed in involving participants from across all four stakeholder categories and operating in both urban and suburban or rural settings across the four nations of the UK (England, Northern Ireland, Scotland and Wales). A total of 29 participants were involved in either focus groups or interviews, as detailed in table 1.

**Table 1:** Breakdown of participants by stakeholder group, mode of participation and country

	Focus group		Interview			Total*
	UK	USA	UK	USA	Other	
Activists	3	3	2	1	0	9
Clinicians	6	0	1	1	0	8
Policy actors	7	1	0	1	0	9
Individuals with lived experience of mpox	0	0	3	0	1 (Germany)	4

* One participant took part in both a focus group and a follow-up interview.

### Data collection

The focus groups were held online via Zoom, with a professional facilitator convening these in the form of adapted ‘deliberative fora’^18^ to prompt discussion and consideration of issues associated with the outbreak. The three focus groups were organised for one stakeholder community each (activists, clinicians and policy actors). Participants ranged from those with moderate experience or influence (eg, trainee doctors, local activists) to senior or high-level stakeholders with potentially regional or national roles (senior policy actors, clinical consultants, heads of large activist organisations). Emphasis was placed on facilitating the focus group as a space where every participant could contribute meaningfully. Focus groups allowed participants to exchange views and generated contrasts between participants’ experiences or contexts which triggered further discussion, although the tight time frame for discussions with busy participants placed limits on the exchanges that could take place between participants. Interviews were conducted with individuals who were unable to attend a focus group or, in one case, with a focus group participant who wished to add additional comments. No focus group was organised for participants with LEM and, instead, interviews were conducted given the privacy concerns and stigma these participants might have experienced.

The groups were organised around a small set of broad questions about experiences of the mpox outbreak, including the use of social media (eg, ‘How did you use social media in the response? What went well?’), and open discussion (focus group schedule available as online supplemental file). Participants’ were requested to limit their opening contributions to 3 min, but they could otherwise speak as many times as they wished. As the groups progressed, follow-up questions on emerging themes were asked. Interviews were conducted with a broad topic guide developed to attain specific detail and experience of the mpox response from participants. On average, focus groups lasted 90 min and interviews lasted 45 min.

### Analysis

Data were subjected to framework analysis^19^ combining both a deductive approach (based on expected codes and categories of interest) and open-coding conducted by two researchers in a subset of three transcripts to develop a coding framework. Broad categories were developed in relation to areas of substantive focus: healthcare, vaccination, communications, stigma, experiences and other. These aligned with the study research aims and were informed by initial encounters with the data. The data were also organised in relation to: lessons learnt, policy recommendations and future research. Each transcript (from focus groups or interviews) was coded by two researchers independently. A face-to-face workshop was held in London in March 2023 where researchers shared their preliminary analyses, with the analysis later extended by the first author, with key initial conclusions discussed and later concretised asynchronously.

### Public and patient involvement

This work was funded under an Economic and Social Research Council (ESRC) urgency response grant between October 2022 and March 2023. The short timeline precluded formalised involvement of people with LEM in project design. Staff from UKHSA were directly involved as team members in the project, and several team members also drew on lived and professional experience engaging with MSM around health and community life (including in relation to mpox).

## Findings

Across stakeholder groups, participants described how social media had played a key role in their experiences of responding to mpox, with participants consistently mentioned Twitter as the most relevant platform. They highlighted the benefits of social media for: disseminating relevant information, allowing for collaboration and advocacy, and tackling stigma. However, they also evidenced how social media had become problematic due to reliance on pre-existing networks, patterns of exclusion and the proliferation of inaccurate or misleading information. These findings are summarised in table 2.

**Table 2:** Summary of findings

Experiences of using social media in mpox responses in the UK	
Benefits	Drawbacks
Dissemination of relevant information	Inaccurate or misleading information
Collaboration and advocacy	Reliance on pre-existing networks
Tackling stigma	Patterns of exclusion

### Benefits of social media use

#### Dissemination of relevant information

In the UK, early vaccine distribution took place through mass walk-in vaccinations clinics. A long-time HIV activist in the UK who had become a leading voice in the mpox response explained that social media had allowed clinics to ‘get the word out’ about vaccination opportunities, and argued that:

if the massive lines for our at-risk communities to get vaccinated are not an example of how social media got the word out and got people in line and ready, then I don’t know what is. (FG1P17)

This view of social media was reinforced by a senior policy actor who commented that ‘social media was really important, it was the way we shared most of our messages with communities’ (IP1). Activist stakeholders also explained how they relied on their personal social media presence to disseminate information about mpox. For example, one participant who, before mpox, had become a self-labelled ‘medical influencer’ around HIV and gay men, explained how:

as a healthcare worker living with HIV, I did feel that I had built the networks and that they were prepared for something like this. This facilitated a lot the transfer of information with other healthcare workers and with communities. (FG1P20)

#### Collaboration and advocacy

Twitter and other platforms also facilitated a degree of collaboration to develop messages and generated community advocacy. One clinician working at the front line of mpox care commented how social media had allowed their local sexual health clinic to engage with community partners:

we had informal links with quite a few kinds of community members who are very active on social media, so we messaged them with information for them to share. (FG2P1)

The involvement of individual social media profiles often relied on informal activities and personal networks, as opposed to involvement being carefully designed and managed by one or more institutional actors. As one activist described:

big media and government were getting it kind of wrong […]. The queer online community was ready for this. We mobilized everybody and went into action online. (FG1P19)

Social media also enabled communities to advocate for better funding and improved responses. For example, a clinician explained how vaccine clinics attendees often turned to social media to complain about long waiting times outdoors and how these complaints were, in turn, picked up by local or national media and put pressure on the vaccination roll-out.^20^

#### Tackling stigma

Participants with LEM argued that social media also allowed them to tackle feelings of stigma at the personal level. One participant who had been admitted to hospital with acute mpox-related symptoms in the UK explained how he had felt that:

there was a lot of shame or embarrassment associated with […] diagnosis. So I went on this militant activist attack and went very public with it, trying to remove stigma by sharing it on social media. […] That helped reduce feelings of stigma. (LEMP11)

One activist described how, on social media,

You’d see people with symptoms. You’d see people actually talking about their own experiences, and I feel people appreciated seeing what’s was really happening. People felt more comfortable with that information. (IP2)

For this participant, and others, social media became a platform to share experiences and communicate with others in ways that tackled stigma and its associated isolation and shame.

### Drawbacks of social media use

#### Reliance on pre-existing networks

The success of using social media to disseminate information relied on the availability of pre-existing social networks and audiences. Their absence, some participants noted, precluded any effective use of social media. For example, one clinician working in a sexual health clinic in the north of England with less than 1000 followers on their official institutional Twitter account explained how:

not having a massive audience on Twitter, like [a large flagship sexual health clinic in the UK] has made it difficult to put out our own messages. We did put some things on social media but it is quite hard to reach people so we mostly retweeted stuff other people put out. (IP4)

That is, compared with the Twitter account for 56 Dean Street (London’s flagship sexual health clinic, with almost 15 000 Twitter followers) the participant’s local clinic lacked the audience to use Twitter as an effective tool in their local response. Furthermore, this clinician—and others—also commented how the competing priorities in the immediate response to the outbreak (including patient care and policy development) made it difficult for organisations to find capacity to work towards rapidly increasing social media audiences; as one focus group participant commented:

we were so busy with seeing patients, writing protocols and pathways, that often communication on social media was an afterthought. (FG2P1)

Limits on capacity in turn limited the degree to which social media might be used to expand connections and networks, and proliferate messages more widely. Instead, organisations had to rely on pre-existing social media networks to efficiently communicate information to communities.

#### Patterns of exclusion

One policy actor stakeholder in the UK explained how relying on community members to disseminate messages to their networks via social media

entrenched inequality because people who were already connected could find the information they needed but others, who wouldn’t be looped in those networks, wouldn’t think it was relevant to then. (FG3P22)

That is, messages disseminated by community members or organisations via social media would oftentimes only reach people already engaged in certain conversations; for instance, people living in the same urban area, or partaking in the same hobbies or social groups (such as clubbing or LGBT+ activism). Furthermore, as one activist reflected, relying on social media as the main communication tool

created disparities because there are a lot of people that still don’t have phones or a place to access the internet. (FG1P11)

Through such digital exclusions, social media campaigns around mpox ‘privileged people that were already part of communities’. By this phrase, the stakeholder referred to already well-served communities in terms of sexual health, such as urban, white, middle-class gay men, at the expense of other groups (minoritised ethnic communities, people living in rural settings, migrants, homeless people and more).

#### Inaccurate or misleading information

At the same time, social media also facilitated the appearance of an ‘infodemic’, which the WHO defines as: ‘too much information including false or misleading information in digital and physical environments during a disease outbreak’.^17^ This meant that both accurate and misleading or inaccurate information was widely spread and that, often, information was foregrounded by platform algorithms that was unhelpful or irrelevant.^3 12^ For example, several participants commented how, in mid-July, as mpox was spreading rapidly in Spain, a tweet from a supposed doctor who pictured a man with mpox lesions riding the Madrid underground rapidly became ‘viral’ worldwide. However, it later emerged that the man pictured was suffering from neurofibromatosis, not mpox. Online pundits and news outlets latched onto impactful but improvable, inaccurate, or outright false stories like this or, for instance, the potential for mpox to become airborne.^21^ This focus was often at the expense of accurate, evidence-based reporting on existing knowledge about mpox transmission or community needs.

## Discussion

Our study contributes to understanding the use of social media in the response to mpox in the UK. It investigates the experiences of four stakeholder communities: activists, clinicians, policy actors and individuals with LEM. Our study highlights the benefits and drawbacks experiences by these communities when using social media in the immediate response to mpox.

Clinician and activist stakeholders described how social media had been useful to swiftly disseminate relevant information about mpox, including information about vaccination opportunities. They also highlighted that online platforms had facilitated a degree of collaboration with community members to engage in sharing or creating information. It is worth noting that, in the experience of our participants, collaboration with community members (whether individuals or organisations) in the UK, relied on informal networks and contacts and were not systematically structured (or funded). By point of contrast, in the USA, for instance, the US Government adapted a strategy from HIV work known as ‘trusted messengers’, by which community ‘partner organisations’ or individual popular opinion leaders would be identified and collaborations set up to develop and deliver bespoke messages to their communities^16^ (such as during the Mid-Atlantic Leather Weekend^21^).

Finally, social media also provided a space for communities to advocate for better responses from institutions and for individuals with LEM to engage in activities (such as sharing information about their diagnoses) that helped them tackle the stigma experienced from living with mpox. These findings support existing literature that argues that social media platforms may be spaces where individuals obtain and provide social support^10^ or build communities.^13^ This role of social media is particularly relevant when individuals navigate conditions that are stigmatised, such as mpox. In these cases, social media may become a space where people who ‘may be reluctant to ask healthcare providers, family members, or peers about medical conditions can turn […], for information and discussion about health topics that are important to them’.^9^

At the same time, however, across all stakeholder groups, participants in this research evidenced how social media also presented important drawbacks. Stakeholders, particularly clinicians, suggested that effectively mobilising social media to disseminate information swiftly relied on the availability of pre-existing networks and audiences. In turn, this often also led, as activist stakeholders articulated, to the exclusion of already-underserved communities. This is in line with existing literature that suggests that, as the ‘digital divide’ narrows and digital communications becomes more prevalent, there is an increasing overlap between digital exclusion (an unequal access or capacity to use digital tools) and other forms of vulnerability and exclusion,^22^ generating a ‘digital underclass’.^23^ This is particularly troubling, as Seifert suggests, during periods of heavy reliance on digital tools for communication and health promotion,^24^ such as COVID or mpox.

At the same time, participants across stakeholder groups also stated that, in social media, accurate and relevant information was often positioned next to inaccurate or misleading information, generating an ‘infodemic’.^11^ This pattern has already been well identified in relation to other health conditions, such as COVID or human papillomavirus.^25 26^

### Applicability beyond the UK

The findings of this research are focused on the UK context. The involvement of international stakeholders helped to provide points of contrast and illustrate alternative responses to mpox as part of the broader research, sharpening our analysis and recommendations. The UK context is characterised by significant fragmentation of provision and the existence of health services which are free at the point of use across its four nations.^27^ This shaped possible forms of collaboration across stakeholder communities in ways which might differ in other healthcare contexts. Nevertheless, fragmentations of healthcare systems is not unusual beyond the UK, and so the lessons learnt about the challenges of this for health promotion are likely to be relevant elsewhere. Existing research has evidenced that the use of social media within public health is an existing concern in contexts where these platforms are widely used.^7 8 10 11 15^ This work has already highlighted that the role of social media in disseminating information, allowing people to tackle stigma. Similarly, researchers have also evidenced the problems of misinformation and exclusion within social media platforms. Therefore, it is likely that these findings are relevant beyond the UK to other settings where social media are similarly used. Our research is less applicable in national contexts where sex between men is illegal or very highly stigmatised, reducing the likelihood of statutory healthcare actors fostering the kinds of collaborations we recommend. This underscores the enduringly damaging health implications of stigma and social and legal injustice.

### Limitations

This project conducted rapid research during a funded period of 6 months between October 2022 and March 2023 to capture ongoing responses to mpox and inform policy in preparation for potential future outbreaks. The limited duration of the project (which is particularly relevant for qualitative research) and the fact that it was conducted while the outbreak was still ongoing mean that this research has several limitations. First, while participants from activist, clinical and policy communities represented wide-ranging experience and expertise, they were mostly white. This is possibly a result of the rapid snowball recruitment relying on the researchers’ personal networks (the research team were all white, and largely male). Second, only a small number of participants with LEM could be recruited during the project time frame. At least two other studies on mpox were being conducted with this group by other teams in the UK—potentially leading to community fatigue. Despite this, the sample of participants recruited among those with LEM for interviews included people of a variety of ethnic backgrounds and ages—although all identified as gay men. Third, the project employed a cross-sectional methodology (focus groups and interviews at a particular point in time), but requested participants to discuss their experiences of the mpox outbreak during the previous months. If it had been possible, a longitudinal methodology with sequential moments of data collection might have better captured differences in social media use across time. Finally, the qualitative discussions were less dynamic than is generally desirable in focus group research, perhaps reflecting the online nature of the data collection (itself essential due to the geographic distances between participants and challenges in finding a time to collectively meet). We do not see the use of a qualitative methodology per se as a limitation, however: qualitative research is well established in the field of health research^28^ and it can lead to very valuable and informative findings in the midst of public health outbreaks.^29^ The combination of focus groups with interviews allowed activists, clinicians and policy actors to engage with each other and contrast their experiences and contexts while interviews allowed for more privacy among people with LEM.

## Conclusions and recommendations

Our research suggests that effective mobilisation of social media in the response to mpox relied on the availability of pre-existing audiences or networks in those platforms. Those participants who described either themselves or their organisations having been able to effectively disseminate messages through social media were also the ones who had a pre-existing audience in those platforms before the outbreak. On the contrary, the absence of those audiences made it difficult—when not impossible—for organisations to reach their intended publics via social media.

Furthermore, participants’ experiences point to concerning dynamics of exclusion: our data suggests that responses to mpox on social media not only relied on pre-existing networks (as we just argued) but went further to entrench those audiences. That is, those individuals who had not partaken in given conversations or engaged in specific online platforms before mpox took place were often excluded from information sharing or collaboration during the outbreak—reinforcing exclusionary trends.

Based on our analysis, we recommend that:

1. Preparedness for outbreaks of infectious diseases such as mpox must consider the role of social media as key tools for collaborating on message development and the dissemination of information.
2. Organisations should allocate funding to developing their social media skills and audiences at a strategic level. This may include developing content, identifying partners, or building relationships that may be deployed during an outbreak.
3. Special attention should also be paid to ensuring that audiences and collaboration are built that promote inclusion of underserved groups.

We argue that these recommendations should be acted on before an epidemic outbreak occurs since what our participants across communities emphasised time and again for a range of examples (extending far beyond social media per se), was that, by the time an outbreak happens, it is already too late to develop the infrastructures needed.

## supplementary material

10.1136/bmjph-2023-000407 online supplemental file 1

## Data Availability

Data sharing not applicable as no datasets generated and/or analysed for this study.

## Footnotes

Since July 2023, Twitter has been renamed ‘“X’”. However, since this research was conducted between October 2022 and March 2023, and since the name ‘“Twitter’” remains in common use, this paper employs the name ‘“Twitter’” throughout, except in the abstract.
