# Supplementary material for: Social media as a public health tool during the UK mpox outbreak: a qualitative study of stakeholders’ experiences
Source: BMJ Public Health. 2023 Oct 25;1(1):e000407. doi: 10.1136/bmjph-2023-000407 (PMC11812727; doi:10.1136/bmjph-2023-000407)
Supplement: online supplemental file 1 [file bmjph-1-1-s001.pdf]

Supplementary file 1: Focus group and interview guide

USING SOCIAL MEDIA AS A PUBLIC HEALTH TOOL DURING THE MPOX OUTBREAK IN THE UK: A QUALITATIVE STUDY OF STAKEHOLDERS' EXPERIENCES AND LESSONS

Garcia-Iglesias, Jaime, Tom May, Martyn Pickersgill, Jeremy Williams, Maurice Nagington, Sophie Buijsen, Ciara J. McHugh, and Jeremy Horwood.

Focus group guide (activists/clinicians/policy actors)

All focus groups will start by welcoming participants and re-stating the terms of the consent form.

These will follow, roughly, this structure:

Firstly, thank you all for your time and your interest in talking to us today. We want to start by asking if you have any questions regarding the focus group information we sent prior to today's conversation?

Great, just so we're on the same page:

- We need to inform you that this focus group is being voice-recorded, but that it will edited for anonymity. Your responses and your participation will not be made public in any way.
- We understand that we may touch upon some sensitive issues. At any point, you have the right to ask for a pause or to leave. We will also have a breakout room available if you prefer.

Do you understand these two things?

Today we want to talk about your experiences of monkeypox as activists/clinicians/policy actors.

This is part of a project we're doing to understand what the social aspects of the response to the monkeypox outbreak were, with a focus on community organising, activism, and how social media might have influenced these. However, please feel free to go on a tangent—that's frequently where the most interesting stuff comes up!

For the sake of the recording, we ask that you speak one at a time. You can use the raise-hand option at the bottom of your screens, text us on the chat, or just raise your hand. Today we have a facilitator who will support this focus group.

[The facilitator introduces themselves. Each of the researchers present also introduces themselves and provides a very brief background.]

Are there any questions before we start?

Initial questions to generate discussion:

- Can you recall when you first heard or knew about the monkeypox outbreak in 2022?
- Can you tell us how your organization/service/department started to think about it? How did they react to the news? Whom did you talk to about the outbreak? How did this change as time went by?

- Did you talk with other people outside from work about it? Did you find that friends or family members would ask you information about it?
- How did you (if you did) use social media during this period? What problems did you encounters? And benefits?
- How do you feel about the information available to the public? Can you share any examples of great information campaigns? And of bad ones? Did you have any role in developing information for people?
- In terms of the people you talk to during your work (patients, clients, etc.), how well informed were they? Do you have a sense of where they might have gotten the information? What kinds of people seemed to be better informed or worse informed?
- Overall, how do you feel your community responded? Do you have any insight into the way other communities responded? What was the same/different? Are there any communities that did better than others? What about people who are not MSM?

As the focus group comes to an end, it will conclude with:

We thank you for taking the time to reflect on and share your experiences. That's all our questions for now. Before we go:

- How did you feel throughout the focus group?
- How can we improve it?
- Is there anything we didn't talk about that you thought would be discussed?
- Was there anything you found surprising?

Interview guide (participants with lived experience of mpox)

As participants are emailed the link to join the Zoom meeting, they will be reminded of the fact that they can keep their cameras off and also given easy instructions for changing their screen name as they join the session.

The introduction and welcoming will follow these lines:

> Firstly, thank you for your time and your interest in talking to us today. We want to start by asking if you have any questions regarding the information we sent prior to today's conversation?
>
> Great, just so we're on the same page:
>
> - We need to inform you that this conversation is being voice-recorded, but that it will edited for anonymity. Your responses and your participation will not be made public in any way.
> - You may keep your camera on or off throughout. That's totally fine.
> - We understand that we may touch upon some sensitive issues. At any point, feel free to just take a breather. If you would prefer to take a break or just leave the call, that's also totally fine.
>
> Do you understand these things?
>
> Today we want to talk about your experiences of monkeypox. This is part of a project we're doing to understand what were the social aspects of the response to the monkeypox outbreak. Do feel free to go on a tangent—that's frequently where the most interesting stuff comes up!
>
> Are there any questions before we start?

Key questions or discussion points to ask:

> - Can you tell us about how you first heard about monkeypox? When was this? Where did you hear about it? What did you think at the time? Did you talk to people about it?
> - Can you tell us about how you found out you had monkeypox? How was it trying to get a diagnosis? Whom did you speak to about it?
> - How was it being sick with monkeypox? What kinds of support did you get, and from whom?
> - How did you use social media during that time? What social media did you use? Were you 'open' about having monkeypox? Why or why not?
> - Have you been talking to others about your experiences? What kinds of people have you spoken with about it? What were their reactions?
> - Using one work, how did it feel to have monkeypox?

As the interview comes to an end, it will conclude with:

> We thank you for taking the time to reflect on and share your experiences. We know how difficult it may be sometimes to talk about these things, but we're incredibly grateful for your insight. That's all our questions for now.
>
> Before we go:
>
> - How did you feel throughout the interview?

- How can we improve it?
- Is there anything we didn't talk about that you thought would be discussed?
- Was there anything you found surprising?

Just a final note: we will get in touch with you over email to send you a small token of gratitude in the form of a shopping voucher.
